# Assessment of Secondary Sulfate Aqueous-Phase Formation Pathways in the Tropical Island City of Haikou: A Chemical Kinetic Perspective

**DOI:** 10.3390/toxics12020105

**Published:** 2024-01-26

**Authors:** Chen Wang, Li Luo, Zifu Xu, Shuhan Liu, Yuxiao Li, Yuanzhe Ni, Shuh-Ji Kao

**Affiliations:** 1 State Key Laboratory of Marine Resource Utilization in South China Sea, Hainan University, Haikou 570228, China; 2 College of Marine Science and Engineering, Hainan University, Haikou 570228, China; 3 Collaborative Innovation Center of Marine Science and Technology, Hainan University, Haikou 570228, China; 4 State Key Laboratory of Marine Environmental Science, College of Ocean and Earth Sciences, Xiamen University, Xiamen 361104, China; 5 Key Laboratory of Marine Environment and Ecology, Ministry of Education, Ocean University of China, Qingdao 266100, China

**Keywords:** sulfate formation, aqueous-phase reaction, influencing factors, tropical island, Haikou

## Abstract

Sulfate (SO_4_^2−^) is an essential chemical species in atmospheric aerosols and plays an influential role in their physical–chemical characteristics. The mechanisms of secondary SO_4_^2−^ aerosol have been intensively studied in air-polluted cities. However, few studies have focused on cities with good air quality. One-year PM_2.5_ samples were collected in the tropical island city of Haikou, and water-soluble inorganic ions, as well as water-soluble Fe and Mn, were analyzed. The results showed that non-sea-salt SO_4_^2−^ (nss-SO_4_^2−^) was the dominant species of water-soluble inorganic ions, accounting for 40–57% of the total water-soluble inorganic ions in PM_2.5_ in Haikou. The S(IV)+H_2_O_2_ pathway was the main formation pathway for secondary SO_4_^2−^ in wintertime in Haikou, contributing to 57% of secondary SO_4_^2−^ formation. By contrast, 54% of secondary SO_4_^2−^ was produced by the S(IV)+Fe×Mn pathway in summer. In spring and autumn, the S(IV)+H_2_O_2_, S(IV)+Fe×Mn, and S(IV)+NO_2_ pathways contributed equally to secondary SO_4_^2−^ formation. The ionic strength was the controlling parameter for the S(IV)+NO_2_ pathway, while pH was identified as a key factor that mediates the S(IV)+H_2_O_2_ and S(IV)+Fe×Mn pathways to produce secondary SO_4_^2−^. This study contributes to our understanding of secondary SO_4_^2−^ production under low PM_2.5_ concentrations but high SO_4_^2−^ percentages.

## 1. Introduction

Sulfate (SO_4_^2−^) is an important component of water-soluble inorganic ions in fine particulate matter (PM_2.5_, aerodynamic diameter ≤ 2.5 µm), accounting for 44–60% of the mass fraction in PM_2.5_ [1,2,3,4,5]. It is well known that sources of SO_4_^2−^ in the atmosphere include primary emission sources (such as sea salt and dust) and secondary SO_4_^2−^ formation (the oxidation of SO_2_ to SO_4_^2−^ in the atmosphere), with the latter being the dominant contributor of fine particulate SO_4_^2−^ [6,7]. Atmospheric secondary SO_4_^2−^ formation pathways include gas-phase reactions, aqueous-phase reactions in aerosols or clouds, and heterogeneous reactions on aerosol surfaces (Figure S1). The gas-phase reaction is the oxidation of SO_2_ by OH· to produce gaseous sulfuric acid (H_2_SO_4(g)_) [8]; subsequently, gas-phase H_2_SO_4(g)_ reacts with alkaline substances (such as NH_3(g)_ and CaCO_3_) to produce particulate SO_4_^2−^. Aqueous-phase reactions include the generation of S(IV) (S(IV) = SO_2_·H_2_O + HSO_3_^−^ + SO_3_^2−^) and the oxidation of S(IV) by NO_2_, H_2_O_2_, O_3_, and O_2_ catalyzed by transition-metal ions (TMIs, e.g., Fe(III) and/or Mn(II)) to form secondary SO_4_^2−^. The heterogeneous reactions are generally referred to as the direct oxidation of SO_2_ to SO_4_^2−^ on aerosol surfaces [9,10]. Among these formation pathways, aqueous-phase reactions are thought to be the most important reactions for secondary SO_4_^2−^ formation and have attracted the most attention.

Due to strong public concerns about air pollution in China and the important contribution of secondary SO_4_^2−^ to PM_2.5_, the aqueous-phase formation of secondary SO_4_^2−^ in aerosols or cloud/fog droplets has received extensive interest in the past decades in air-polluted areas in China. Based on WRF-CMAQ analysis, Cheng et al. reported that S(IV)+NO_2_ was the dominant pathway for secondary SO_4_^2−^ formation under a pH ranging from 5.4 to 6.2 during haze pollution periods in Beijing [11]. By combining observational datasets with an observation-based model for simulating secondary inorganic aerosol, Xue et al. found that the S(IV)+NO_2_ pathway was prevalent during haze–fog events in Shanghai, Nanjing, and Guangzhou [12]. By combining atmospheric measurements and laboratory simulations, Wang et al. reported that the aqueous oxidation of SO_2_ by NO_2_ was a key pathway for secondary SO_4_^2−^ formation during air-polluted periods [13]. By using online observations and developing an improved solute intensity-dependent chemical thermodynamics and kinetics model, Gao et al. reported that the S(IV)+H_2_O_2_ pathway dominates sulfate formation in Tianjin during haze pollution periods [14]. By coupling a laboratory simulation and a state-of-the-art multiphase model, Song et al. suggested that the TMI-catalyzed pathway was the most important one for secondary SO_4_^2−^ formation in North China [15]. Although much research has been conducted on the secondary SO_4_^2−^ aqueous-phase formation rate, previous studies have mainly focused on haze pollution periods [11,12,13,14,15,16,17]. Few studies have explored the secondary SO_4_^2−^ aqueous-phase formation rate under the condition of low PM_2.5_ concentration. In particular, in China, after the implementation of the Clean Air Act and the strengthening of the government resolve on air pollution, the PM_2.5_ concentration decreased to low levels. However, the changes in secondary SO_4_^2−^ aqueous-phase formation rates remain unclear.

According to the 14th Five-Year Plan of Hainan Province, the annual average PM_2.5_ concentration in Hainan Province should be lower than 11 μg m^−3^. However, the average PM_2.5_ concentration in recent years in Haikou has been 16.7 μg m^−3^, especially in winter, during which the PM_2.5_ concentration can be as high as 30 μg m^−3^. As a pilot zone for ecological conservation and a free trade port with Chinese characteristics, Hainan Province still has the daunting task of managing its atmospheric PM_2.5_ to establish its ecological environment as a world leader. Previous observations in Haikou in 2011–2012 found that SO_4_^2−^ was the most abundant inorganic ion in PM_2.5_ [18], but the formation mechanisms and sources of secondary SO_4_^2−^ formation remain undocumented. The multi-resolution emission inventory for China (MEIC) reported that the total emissions of SO_2_ in Hainan Province in 2020 was 36,114 t, and industry emissions were thought to be the dominant source [19,20]. However, gas-phase SO_2_ is not equal to SO_4_^2−^ in PM_2.5_. If one wants to deeply understand the accumulation of secondary SO_4_^2−^ in PM_2.5_, the first step is to clarify secondary SO_4_^2−^ chemical formation mechanisms. In this study, we collected PM_2.5_ samples from September 2021 to August 2022 and analyzed the concentrations of water-soluble inorganic ions, and water-soluble Fe and Mn. This study aims to (1) calculate the formation rate of secondary SO_4_^2−^ using chemical kinetic models and (2) explore the influences of ionic strength and pH on secondary SO_4_^2−^ formation rates.

## 2. Methods

### 2.1. Sampling

The sampling location was set on a rooftop (24 m above the ground) of the State Key Laboratory of Marine Resource Utilization in the South China Sea, Hainan University (20°06′ N,110°32′ E), Haikou, China (Figure S2). PM_2.5_ samples were collected using a high-volume sampler equipped with a PM_2.5_ cascade impactor and quartz filters (TISSUQUARTZ-2500QAT-UP, PALL Corporation, New York, NY, USA). The sampling duration was 24 or 48 h. Before sampling, all filters were combusted at 450 °C for 6 h in a muffle furnace. After sampling, the filters were stored in clear Ziplock bags and immediately refrigerated at −20 °C. A total of 200 PM_2.5_ samples were obtained from 1 September 2021 to 30 August 2022. We divided the sampling periods into four seasons (autumn (September to November 2021), winter (December 2021 to February 2022), spring (March to May 2022), and summer (June to August 2022)). The meteorological parameters (temperature and RH) were downloaded from http://www.weather.com.cn (accessed from 1 September 2021 to 30 August 2022), and there were no significant seasonal variations for RH in contrast with obvious seasonal differences for temperature (Figure S3a). Hourly air pollutants (such as PM_2.5_, PM_10_, SO_2_, NO_2_, O_3_, CO, Figure S3b–d) were obtained from an air quality monitoring station (https://map.zq12369.com; accessed from 1 September 2021 to 30 August 2022) located 300 m from our sampling site.

### 2.2. Chemical Analysis

#### Analysis of Water-Soluble Ions, and Water-Soluble Fe and Mn

One-quarter of the filters were placed in clear 50 mL centrifuge tubes, and 30 mL of Milli-Q water (18.2 Ω) was added to immerse the filters, followed by ultrasound for 30 min and then rest for 30 min at room temperature. The extracts were filtered through a membrane filter (0.22 µm). Then, 5 mL extracts were used for water-soluble ion measurements and 9.5 mL extracts were used for water-soluble Fe and Mn analysis. The water-soluble ions (Na^+^, NH_4_^+^, K^+^, Mg^2+^, Ca^2+^, Cl^−^, NO_3_^−^, SO_4_^2−^) were analyzed by Ion Chromatography (DIONEX AQ-1100, Dionex Aquion RFIC ThermoFisher, CS12A for cations, and AS22 for anions). The anionic eluents comprised 4.5 mM Na_2_CO_3_ mixed with 1.4 mM NaHCO_3_, and the cationic eluents comprised 20 mM methanesulfonic acid (MSA). The concentration of each ion in the blank was subtracted from the measured ion concentrations of each sample to remove possible contamination during the test.

The water-soluble Fe and Mn were analyzed by inductively coupled plasma optical emission spectrometry (Agilent 5100, Agilent Technologies Inc., Santa Clara, CA, USA). Before analysis, 0.5 mL of pure nitric acid (65%) was added to the 9.5 mL extracts. All concentrations were corrected for background concentrations with duplicate filter blanks. The detection limits for water-soluble Fe and Mn were 0.1 µmol L^−1^ and 0.11 µmol L^−1^, respectively (3 × blank standard deviation). The accuracy of instrument testing was ensured by inserting a quality control solution every 10 samples, and the 44 repeat analyses of quality control solutions for water-soluble Fe and Mn were 100 ± 0.3 μg L^−1^.

### 2.3. Data Analysis

#### 2.3.1. The Concentration of nss-SO_4_^2−^

The concentration of nss-SO_4_^2−^ in PM_2.5_ is as follows:

[nss-SO_4_^2−^] = [SO_4_^2−^] − [ss-SO_4_^2−^] (1)

[ss-SO_4_^2−^] = 0.252 × [Na^+^] (2)

where ss-SO_4_^2−^ represents sea-salt SO_4_^2−^, SO_4_^2−^ and Na^+^ are calculated using the unit of mass concentration (µg m^−3^) of water-soluble ions, and 0.252 is the seawater SO_4_^2−^/Na^+^ mass ratio [21,22]. 

#### 2.3.2. Aerosol Water Content (AWC), Aerosol pH, and Ionic Strength

The AWC, aerosol pH, and ionic strength were calculated using ISORROPIA II. ISORROPIA II calculates the compositions and phase state of Na^+^-K^+^-Ca^2+^-Mg^2+^-NH_4_^+^-SO_4_^2−^-NO_3_^−^-Cl^−^-H_2_O. This model (ISORROPIA v2.1) was developed by Athanasios Nenes and Christors Fountoukis at the University of Miami, Carnegie Mellon University, and the Georgia Institute of Technology. It has two input units (µmol m^−^^3^ air and µg m^−^^3^ air), two modes (forward and reverse modes), and two aerosol states (stable and metastable states). In this study, we chose µg m^−^^3^ air, forward mode, and metastable state and then input the concentration of water-soluble inorganic ions, RH, and thermodynamic temperature to calculate the AWC, aerosol pH, and ionic strength. Detailed information about ISORROPIA II can be in the work of Fountoukis and Nenes [23]. ISORROPIA II has been widely used to calculate AWC, aerosol pH, and ionic strength [14,15,17,24,25].

#### 2.3.3. The Secondary SO_4_^2−^ Formation Rates in Aqueous-Phase Chemistry

The aqueous-phase formation steps of secondary SO_4_^2−^ include the transformation of SO_2_ into S(IV) and the oxidation of S(IV) into secondary SO_4_^2−^ by various oxidants. The detailed calculations are as follows. 

##### The S(IV) Concentration

Assuming that gas-phase X is in equilibrium with aqueous X in aerosol water, the concentration of dissolved X ([X(aq)], (M)) can be expressed using Equation (3)

[X(aq)] = H(X)p(X)(3)

where X represents the concentration of SO_2_ or other oxidants (NO_2_, H_2_O_2_, and O_3_) and p(X) is the partial pressure of X in the atmosphere (atm). H(X) represents Henry’s law constant for X, and the unit of Henry’s law constant is M^−1^ atm^−1^. 

The concentrations of SO_2_·H_2_O, HSO_3_^−^, and SO_3_^2−^ in aerosol water are given by Equations (4)–(6) [26],

(4) $${[{SO}_{2}{\cdot H}_{2}O]=H}_{{SO}_{2}}p_{{SO}_{2}}$$

(5) $$\left[{HSO}_{3}^{-}\right]=\frac{H_{{SO}_{2}}K_{s1}p_{{SO}_{2}}}{{[H}^{+}]}$$

(6) $$\left[{SO}_{3}^{2-}\right]=\frac{H_{{SO}_{2}}K_{s1}{K_{s2}p}_{{SO}_{2}}}{{{[H}^{+}]}^{2}}$$

where H_SO2_ is Henry’s law constant for SO_2_ (M^−1^ atm^−1^), [H^+^] = 10^−pH^ M, and pH is calculated via ISORROPIA II. K_s1_ (M) and K_s2_ (M) are the first and second dissociation equilibrium constants for HSO_3_^−^ and SO_3_^2−^, respectively. The detailed calculations for H_SO2_, K_s1_, and K_s2_ are described in Text S1. 

The total S(IV) (M) (S(IV) = SO_2_·H_2_O + HSO_3_^−^ + SO_3_^2−^) concentration can be calculated using Equation (7), and the mole fractions of SO_2_·H_2_O, HSO_3_^−^, and SO_3_^2−^ to total S(IV) can be calculated using Equations (8)–(10) [26],

(7) $$\left[S\left(IV\right)\right]=H_{{SO}_{2}}p_{{SO}_{2}}\left[1+\frac{K_{s1}}{{[H}^{+}]}+\frac{K_{s1}K_{s2}}{{{[H}^{+}]}^{2}}\right]$$

(8) $$x_{{SO}_{2}\cdot H_{2}O}=\frac{{[SO}_{2}\cdot H_{2}O]}{\left[S\left(IV\right)\right]}={\left(1+\frac{K_{s1}}{{[H}^{+}]}+\frac{K_{s1}K_{s2}}{{{[H}^{+}]}^{2}}\right)}^{-1}$$

(9) $$x_{{HSO}_{3}^{-}}=\frac{{[HSO}_{3}^{-}]}{\left[S\left(IV\right)\right]}={\left(1+\frac{{[H}^{+}]}{K_{s1}}+\frac{K_{s2}}{{[H}^{+}]}\right)}^{-1}$$

(10) $$x_{{SO}_{3}^{2-}}=\frac{{[SO}_{3}^{2-}]}{\left[S\left(IV\right)\right]}={\left(1+\frac{{[H}^{+}]}{K_{s2}}+\frac{{{[H}^{+}]}^{2}}{K_{s1}K_{s2}}\right)}^{-1}$$

#### The Oxidation Rate of S(IV) by NO_2_

The reaction rate of S(IV)+NO_2_ pathway was given by Lee and Schwart [27]:

(11) $$R_{aq(NO_{2})}=k_{S\left(IV\right)+{NO}_{2}}\left[S(IV)\right][{NO}_{2}(aq)]$$

where [S(IV)] (M) can be calculated using Equation (7), [NO_2(aq)_] (M) is the aqueous concentration of NO_2_ in aerosol water and can be calculated using Equation (3). Moreover,

(12) $$k_{{S\left(IV\right)+NO}_{2}}=\frac{k_{{S\left(IV\right)+NO}_{2},low+}k_{{S\left(IV\right)+NO}_{2},high}}{2}$$

where k_S(IV)+NO2_ is the rate constant: when pH < 5, k_S(IV)+NO2,low_ = 1.4 × 10^5^ M^−1^ s^−1^; when pH > 5.8, k_S(IV)+NO2,low_ = 2 × 10^6^ M^−1^ s^−1^ [27]; when pH is between 5 and 5.8, k_S(IV)+NO2,low_ = (23.25 × pH − 114.85) × 10^5^ [10]; when pH < 5.3, k_S(IV)+NO2,high_ = 1.24 × 10^7^ M^−1^ s^−1^; when pH > 8.7, k_S(IV)+NO2,high_ = 1.67 × 10^7^ M^−1^ s^−1^ [28]; and when pH is between 5.3 and 8.7, k_S(IV)+NO2,high_ = (1.26 × pH − 5.70) × 10^6^ [10]. More detailed information can be found in Table S2. The influences of ionic strength on the reaction rate of the S(IV)+NO_2_ pathway can be found in Text S2.

##### The Oxidation Rate of S(IV) by H_2_O_2_

The reaction rate of the S(IV)+H_2_O_2_ pathway was given by Hoffmann and Calvert [29]:

(13) $$R_{aq(H_{2}O_{2})}=\frac{k_{(S\left(IV\right)+H_{2}O_{2})1}\left[H_{2}O_{2}\right][{HSO}_{3}^{-}]}{1+\alpha [H^{+}]}$$

where k_(S(IV)+H2O2)1_ can be estimated using Equation (14) [11,26],

(14) $$k_{\left(S\left(IV\right)+H_{2}O_{2}\right)1}\left(T\right)=k_{\left(S\left(IV\right)+H_{2}O_{2}\right)1}\left(298K\right)exp\left[-\frac{E}{R}\left(\frac{1}{T}-\frac{1}{T_{298K}}\right)\right]$$

When the temperature is 298 K, k_(S(IV)+H2O2)1_(298 K) is equal to 7.45 × 10^7^ M^−1^ s^−1^. E/R (K) is listed in Table S2. α is equal to 13 M^−1^ [14]. [H^+^] (M) is the H^+^ concentration, calculated using ISORROPIA II. [HSO_3_^−^] (M) is the only species of S(IV) that reacts with H_2_O_2_ to form secondary SO_4_^2−^, calculated using Equation (5). [H_2_O_2_] is the mole fraction of H_2_O_2_ (nmol mol^−1^), estimated using an empirical equation [14,30],

(15) $$\left[H_{2}O_{2}\right]=0.1155e^{0.0846T}$$

where T is the ambient temperature (°C). The influences of ionic strength on the reaction rate of the S(IV)+H_2_O_2_ pathway are given in detail in Text S3.

##### The Oxidation Rate of S(IV) by O_3_

The reaction rate of S(IV)+O_3_ pathway was given by Hoffmann and Calvert [29]:

(16) $${R_{aq(O_{3})}=(k}_{(S\left(IV\right)+O_{3})1}\left[SO_{2}\cdot H_{2}O\right]+k_{(S\left(IV\right)+O_{3})2}\left[{HSO}_{3}^{-}\right]+k_{(S\left(IV\right)+O_{3})3}\left[{SO}_{3}^{2-}\right]{)[O}_{3}(aq)]$$

where k_(S(IV)+O3)1_ = 2.4 × 10^4^ M^−1^ s^−1^, and k_(S(IV)+O3)2_ (M^−1^ s^−1^) and k_(S(IV)+O3)3_ (M^−1^ s^−1^) are functions of temperature [11,26],

(17) $$k_{S\left(IV\right)+O_{3}}\left(T\right)=k_{S\left(IV\right)+O_{3}}\left(298K\right)exp\left[-\frac{E}{R}\left(\frac{1}{T}-\frac{1}{T_{298K}}\right)\right]$$

At a temperature of 298 K, k_(S(IV)+O3)2_(298 K) and k_(S(IV)+O3)3_(298 K) are equal to 3.7 × 10^5^ M^−1^ s^−1^ and 1.5 × 10^9^ M^−1^ s^−1^, respectively (Table S2). [SO_2_·H_2_O], [HSO_3_^−^], and [SO_3_^2−^] can be calculated using Equations (4)–(6), respectively. [O_3_(aq)] (M) can be calculated using Equation (3), and Henry’s law constant for O_3_ (H_O3_(298 K)) is equal to 1.1 × 10^−2^ M atm^−1^ (Table S1). The influences of ionic strength on the reaction rate of the S(IV)+O_3_ pathway are detailed in Text S4.

##### The Rate of Fe(III)- and Mn(II)-Catalyzed Oxidation of S(IV) into Secondary SO_4_^2−^

S(IV) oxidation by O_2_ is known to be catalyzed by means of water-soluble Fe(III) and Mn(II) [14]. The rate expression of Fe(III)-catalyzed and Mn(II)-catalyzed oxidation of S(IV) into secondary SO_4_^2−^ is provided in Text S5. The following rates for the synergistic Fe(III)-Mn(II)-catalyzed oxidation of S(IV) (hereafter referred to as S(IV)+Fe×Mn) into *secondary* SO_4_^2−^ were obtained [31]:

pH ≤ 4.2:

(18) $$R_{aq(Fe\times Mn+O_{2})}=k_{(Fe\times Mn+O_{2})1}{\left[H^{+}\right]}^{-0.74}[Fe(III)][Mn(II)][S(IV)]$$

pH ≥ 4.2:

(19) $$R_{aq(Fe\times Mn+O_{2})}=k_{(Fe\times Mn+O_{2})2}{\left[H^{+}\right]}^{0.67}[Fe(III)][Mn(II)][S(IV)]$$

where k_(Fe×Mn+O2)1_ and k_(Fe×Mn+O2)2_ are functions of temperature [14,26]

(20) $$k_{\left(Fe\times Mn+O_{2}\right)}\left(T\right)=k_{\left(Fe\times Mn+O_{2}\right)}\left(297K\right)exp\left[-\frac{E}{R}\left(\frac{1}{T}-\frac{1}{T_{297K}}\right)\right]$$

When the temperature is 297 K, k_(Fe×Mn+O2)1_(297 K) and k_(Fe×Mn+O2)2_(297 K) are equal to 3.72 × 10^7^ M^−2^ s^−1^ and 2.51 × 10^13^ M^−2^ s^−1^, respectively (Table S2). [H^+^] (M) is the H^+^ concentration, calculated using ISORROPIA II, [S(IV)] (M) can be calculated using Equation (7), the concentrations of [Fe(III)] and [Mn(II)] (M) and the influences of ionic strength on the reaction rate of S(IV)+Fe×Mn pathway are detailed in Texts S5 and S6.

##### Mass Transport Limitations Rate

The total reaction rate of oxidation of S(IV) into secondary SO_4_^2−^ (R_H,aq_) is affected by both the rate of chemical reactions (R_aq_) and the rate of limiting mass transfer (J_aq,lim_) in different media and across interfaces. By following Cheng et al. [11],

(21) $$\frac{1}{R_{H,aq}}=\frac{1}{R_{aq}}+\frac{1}{J_{aq,lim}}$$

where R_H,aq_ (M s^−1^) is the total reaction rate of oxidation of S(IV) into secondary SO_4_^2−^ by different oxidants (NO_2_, H_2_O_2_, O_3_, and O_2_). R_aq_ (M^−1^ s^−1^) is the aqueous-phase reaction rate calculated in the Sections dealing with “The Oxidation Rate of S(IV) by NO_2_”,“The Oxidation Rate of S(IV) by H_2_O_2_”, “The Oxidation Rate of S(IV) by O_3_” and “The Rate of Fe(III) and Mn(II) Catalyzed Oxidation of S(IV) into Secondary SO_4_^2−^“. J_aq,lim_ is the limiting mass transfer rate (M s^−1^), the calculating formula of which is detailed in Text S7. 

The final sulfate formation rate was converted to μg m^−3^ h^−1^ units, calculated as follows:

(22) $$R_{a}=3600s\;h^{-1}\times 96g\;{mol}^{-1}\times \frac{AWC}{\rho_{w}}\times R_{H,aq}$$

where 3600 s h^−1^ is the time conversion factor; 96 g mol^−1^ is the molar mass of SO_4_^2−^; AWC is the aerosol water content (mg m^−3^), calculated using ISORROPIA II; ρ_w_ is the density of water, which is 1 kg L^−1^; and R_H,aq_ (M s^−1^) can be calculated using Equation (21).

## 3. Results and Discussion

### 3.1. Seasonal Variations in Water-Soluble Inorganic Ions, Fe and Mn in PM_2.5_

**Figure 1:**
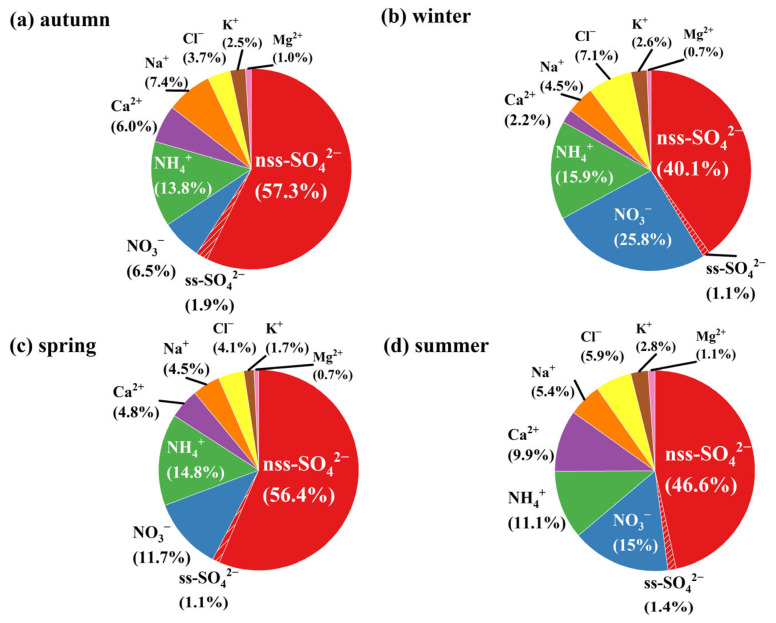
The percentages of water-soluble inorganic ions in PM_2.5_ in autumn (**a**), winter (**b**), spring (**c**), and summer (**d**) in Haikou.

In our observations, SO_4_^2−^ was the most abundant ion, accounting for 41–59% of the total water-soluble inorganic ions in PM_2.5_, and nearly all SO_4_^2−^ was nss-SO_4_^2−^ (Figure 1), indicating that nss-SO_4_^2−^ dominates water-soluble inorganic ionic compositions in PM_2.5_ in Haikou. The nss-SO_4_^2−^ in PM_2.5_ in Haikou has been attributed to secondary sources rather than primary emissions [18]. The highest nss-SO_4_^2−^ concentrations were observed in winter (4.1 ± 1.8 μg m^−3^), and the lowest nss-SO_4_^2−^ concentrations were observed in summer (1.5 ± 0.7 μg m^−3^; see Table S3). Similar seasonal patterns for aerosol nss-SO_4_^2−^ concentrations have been widely reported in many Chinese cities, including Shanghai [32], Chongqing [33], Guiyang [5], Guangzhou [34], and Hong Kong [35]. Higher nss-SO_4_^2−^ concentrations in winter than in summer have been widely attributed to the high SO_2_ emissions and SO_2_ oxidation rates during winter [19,20,36]. NO_3_^−^ and NH_4_^+^ were the secondary abundant ions, accounting for 6.5–26% and 11–16%, respectively, of the total water-soluble inorganic ions in PM_2.5_ (Figure 1). The percentages of other water-soluble inorganic ions (Ca^2+^, Na^+^, Cl^−^, K^+^, and Mg^2+^) to total water-soluble inorganic ions were 16–25% (Figure 1).

In general, Fe in aerosol is entirely sourced from mineral dust, while Mn originates from both mineral dust and anthropogenic activities [37,38]. The concentrations of water-soluble Fe and Mn were 7.4 ± 6.2 and 3.7 ± 2.4 ng m^−3^ in autumn, 15 ± 12 and 6.1 ± 4.4 ng m^−3^ in winter, 12 ± 8.7 and 5.1 ± 2.1 ng m^−3^ in spring, and 4.2 ± 4.1 and 2.8 ± 1.1 ng m^−3^ in summer, respectively. The concentrations of water-soluble Fe in our measurements were lower than those in the summertime in Xi’an (297 ± 78 ng m^−3^) [39] and Singapore (18 ± 2.4 ng m^−3^) [40]; however, the concentrations of water-soluble Mn were higher than those in Singapore (1.1 ± 0.4 ng m^−3^) [40]. In winter, water-soluble Fe concentrations in Haikou were lower than those in Beijing (68 ± 46 ng m^−3^), Handan (59 ± 33 ng m^−3^), Zhengzhou (32 ± 20 ng m^−3^), and Hangzhou (24 ± 8.5 ng m^−3^) [41]. The concentrations of water-soluble Mn during wintertime were also lower than those in North China (e.g., 42 ± 17 ng m^−3^ in Baoding and 55 ± 33 ng m^−3^ in Tianjin [42]). The spatial and temporal differences in water-soluble Fe and Mn are influenced by many factors, such as sources, aerosol aging, and aerosol acidity [43]. 

### 3.2. Seasonal Differences in H_2_O_2_, AWC, Aerosol pH, Ionic Strength, Fe(III)×Mn(II) and S(IV) 

**Figure 2:**
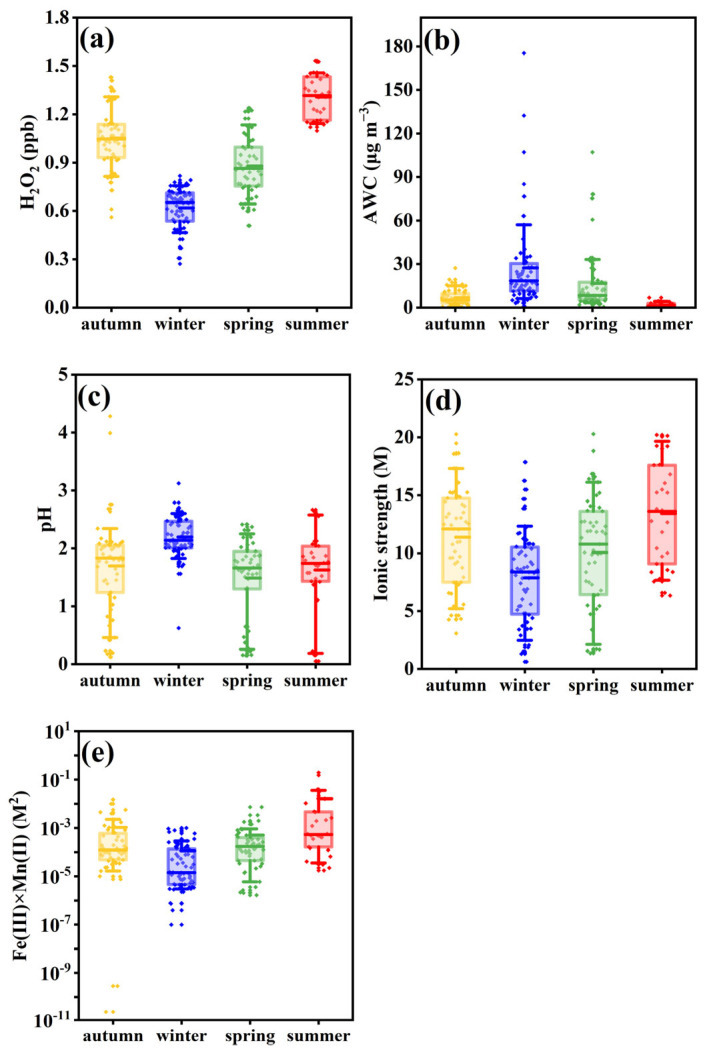
Box plots of seasonal H_2_O_2_ concentrations (**a**), AWC (**b**), pH (**c**), ionic strength (**d**), and Fe(III)×Mn(II) concentrations (**e**). The yellow, blue, green, and red boxes represent autumn, winter, spring, and summer, respectively. The large boxes indicate the interquartile range from the 25th to 75th percentile. The dashed line inside the box indicates the average value. The solid line indicates the median value and whiskers indicate the 10th and 90th percentiles.

The concentrations of H_2_O_2_ ranged from 0.3 to 1.5 ppb, displaying significant seasonal variations, with the highest values in summer (1.3 ± 0.1 ppb) and the lowest values in winter (0.6 ± 0.1 ppb, Figure 2a). Our calculated H_2_O_2_ concentrations within previous reports ranged from 0.03 ± 0.02 ppb to 2.2 ± 0.03 ppb (see Figure S4). The concentrations of AWC ranged from 0.2 to 175 μg m^−3^ (Figure 2b), with the highest concentrations in winter (27 ± 30 μg m^−3^) and the lowest concentrations in summer (1.7 ± 1.7 μg m^−3^). The ranges and seasonal variations in AWC in our calculations are consistent with previous studies in other cities [44,45].

The pH values spanned from 0.05 to 4.3 across all the observations. Individually, pH values ranged from 0.1 to 4.3, 0.6 to 3.1, 0.2 to 2.4, and 0.05 to 2.7 in autumn, winter, spring, and summer, respectively (Figure 2c). Our calculated pH values agree with the aerosol pH values in Greece (−0.1–3.8) [46], Canada (2.5–5.5) [47], and America (0–2) [48] but are lower than the pH values in Beijing (3.0–4.9,) [49] and Shanghai (4.7–6.6) [50]. The ionic strength levels ranged from 0.6 to 20 M, spanning from 3.1 to 20 M, 0.6 to 18 M, 1.4 to 20 M, and 6.3 to 20 M in autumn (11 ± 4.4 M), winter (7.9 ± 4.0 M), spring (10 ± 4.8 M), and summer (13 ± 4.5 M), respectively (Figure 2d). In autumn, the ionic strength levels recorded in our study are similar to another study in Beijing (11–52 M) [25], but our recorded wintertime and summertime ionic strength levels are lower than those reported in the North China Plain (30–50 M and 20–25 M) [15,42].

Concentrations of Fe(III)×Mn(II) varied from 2.3 × 10^−11^ M^2^ to 1.9 × 10^−1^ M^2^, displaying significant seasonal variations (lower concentrations of Fe(III)×Mn(II) in winter (1.1 × 10^−4^ ± 2.2 × 10^−4^ M^2^) than those in summer (1.6 × 10^−2^ ± 4.4 × 10^−2^ M^2^, Figure 2e)). The Fe(III)×Mn(II) concentrations in our observations are higher than those recorded in Tianjin (8.2 × 10^−18^–2.0 × 10^−6^) [14]. The calculated partitions of SO_2_·H_2_O, HSO_3_^−^, and SO_3_^2−^ (Figure S5) are consistent with a previous study, emphasizing the validity of our calculations for S(IV) [26]. When pH < 2, SO_2_·H_2_O is the main species of S(IV), accounting for 50–100% of total S(IV). When the pH is between 2 and 4.5, 50–100% of S(IV) is in the form of HSO_3_^−^. In our observations, all pH values were lower than 4.5 (Figure 2c); thus, the presence of SO_3_^2−^ in aerosol water can be neglected in our case.

### 3.3. The Aqueous-Phase Formation Rates of Secondary SO_4_^2−^

**Figure 3:**
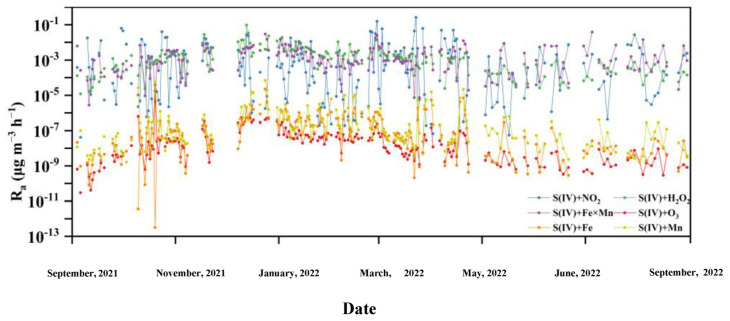
Time series of secondary SO_4_^2−^ formation rates by six different SO_4_^2−^ aqueous-phase formation pathways, made on the first day of a month.

Figure 3 shows the daily secondary SO_4_^2−^ aqueous-phase formation rates by various formation pathways. The results showed that secondary SO_4_^2−^ aqueous-phase formation rates by the S(IV)+NO_2_, S(IV)+H_2_O_2_, and S(IV)+Fe×Mn pathways were 4–7 orders of magnitude faster than the S(IV)+O_3_, S(IV)+Fe, and S(IV)+Mn pathways. Previous studies during Beijing haze periods found that S(IV)+O_3_ was an important pathway for secondary SO_4_^2−^ production when pH > 5.8 [11,51]. As shown in Figure S5, when the pH is between 4.5 and 8, S(IV) partitioning shifts in favor of SO_3_^2−^, and the rate constants for SO_3_^2−^+O_3_ ($k_{\left(S(IV)+O_{3}\right)3}$ = 1.5 × 10^9^ M^−1^ s^−1^) are almost 4–5 orders of magnitude faster than for SO_2_·H_2_O+O_3_ ($k_{(S(IV)+O_{3})1}$) and HSO_3_^−^+O_3_ ($k_{\left(S(IV)+O_{3}\right)2}$ (Table S2), highlighting that the S(IV)+O_3_ pathway is important for secondary SO_4_^2−^ when aerosol pH > 4.5. However, in our observations, the pH range was 0.05–4.3 (Figure 2c), SO_2_·H_2_O and HSO_3_^−^ were the dominant species of S(IV) (Figure S5), and the rate constants of SO_2_·H_2_O+O_3_ and HSO_3_^−^+O_3_ were significantly lower than S(IV)+NO_2_, S(IV)+H_2_O_2_, and S(IV)+Fe×Mn (Table S2); thus, we ignored the S(IV)+O_3_ pathway for secondary SO_4_^2−^ formation. Using model simulations and in-field observations, previous studies also found that secondary SO_4_^2−^ production by the S(IV)+O_3_ pathway was unimportant under conditions with pH < 4.5 [10,11,51,52]. In addition, we also neglected the S(IV)+Fe and S(IV)+Mn pathways for secondary SO_4_^2−^ formation due to their extremely low rate constants (Table S2). Although rate constants k_(Fe +O2)2_ under pH range of 3 and 4.5 were high in the S(IV)+Fe×Mn pathway (Table S2), the extremely low concentrations of Fe(III) (Figure S6) limited secondary SO_4_^2−^ formation by k_(Fe+O2)2_ [Fe(III)]^2^[S(IV)] under pH range from 3 to 4.5. The aqueous-phase formation rates of secondary SO_4_^2−^ by S(IV)+Mn reactions also displayed low rates, which was attributed to the lowest rate constant of k_(Mn+O2)_ (Table S2). Therefore, we did not consider the S(IV)+O_3_, S(IV)+Fe, and S(IV)+Mn pathways due to their low contributions to secondary SO_4_^2−^ production.

### 3.4. S(IV)+NO_2_ Pathway Formation Rates and their Influencing Factors

**Figure 4:**
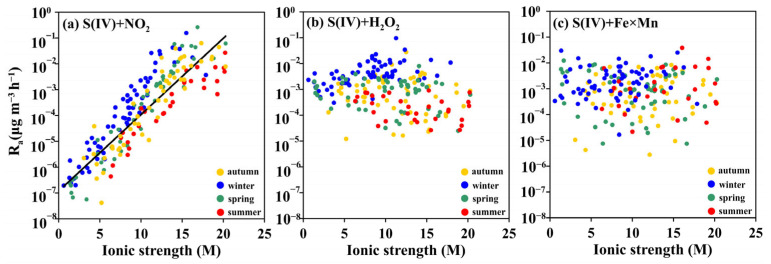
The effect of ionic strength on secondary SO_4_^2−^ formation rates by the S(IV)+NO_2_ (**a**), S(IV)+H_2_O_2_ (**b**), and S(IV)+Fe×Mn (**c**) pathways (yellow, blue, green, and red dots represent autumn, winter, spring, and summer, respectively).

The secondary SO_4_^2−^ formation rates by the S(IV)+NO_2_ pathway exhibited ranges of 4.2 × 10^−8^–6.3 × 10^−2^ µg m^−3^ h^−1^ in autumn, 1.9 × 10^−7^–1.6× 10^−1^ µg m^−3^ h^−1^ in winter, 5.6 × 10^−8^–2.6 × 10^−1^ µg m^−3^ h^−1^ in spring, and 4.3 × 10^−7^–2.7 × 10^−2^ µg m^−3^ h^−1^ in summer (Figure 3). Our calculated secondary SO_4_^2−^ formation rates by the S(IV)+NO_2_ pathway in autumn and winter were lower than those from previous studies in Beijing (2.0 × 10^−4^–5.9 µg m^−3^ h^−1^) [17] and Tianjin (~6.0 µg m^−3^ h^−1^) [14]. In addition to the effect of substrate (S(IV) and NO_2_) concentrations, ionic strength was the main factor that modified secondary SO_4_^2−^ formation by the S(IV)+NO_2_ pathway. As shown in Figure 4a, when ionic strength increased, the secondary SO_4_^2−^ formation rate by the S(IV)+NO_2_ pathway increased, which is consistent with previous studies [10,53]. Two possible mechanisms have been proposed to explain secondary SO_4_^2−^ formation by the S(IV)+NO_2_ pathway. The first is an oxygen atom transfer reaction [28]:

2NO_2_ + SO_3_^2−^ ↔ (O_2_N−SO_3_−NO_2_)^2−^ (R1)

(O_2_N−SO_3_−NO_2_)^2−^ + OH^−^ ↔ (HO−SO_3_−(NO_2_)_2_)^3−^ (R2)

(HO−SO_3_−(NO_2_)_2_)^3−^ + OH^−^ ↔ NO^2−^ + SO_4_^2−^ + H_2_O (R3)

The second is an electron transfer reaction, followed by the reaction of hydroxyl radical with a sulfite radical [54]:

NO_2_ + SO_3_^2−^ → NO_2_^−^ + SO_3_^•−^ (R4)

OH^•^ + SO_3_^•−^ → H^+^ + SO_4_^2−^ (R5)

For both mechanisms, the controlling step is the reaction of an ion with a neutral molecule [11]. The positive trends between the chemical rate constant k_S(IV)+NO2_ and ionic strength (Figure S7) as well as the secondary SO_4_^2−^ formation rates by the S(IV)+NO_2_ pathway and ionic strength (Figure 4a) support the fact that increasing ionic strength enhances secondary SO_4_^2−^ formation rates by the S(IV)+NO_2_ pathway [14,53,55]. 

### 3.5. S(IV)+H_2_O_2_ and S(IV)+Fe×Mn Pathway Formation Rates and Their Influencing Factors

The secondary SO_4_^2−^ formation rates by the S(IV)+H_2_O_2_ pathway in winter (3.0 × 10^−4^–9.5 × 10^−2^ µg m^−3^ h^−1^) were higher than those in summer (2.6 × 10^−5^–9.1 × 10^−4^ µg m^−3^ h^−1^, Figure 3), which is consistent with a previous study in Tianjin, revealing higher values in winter than summer [14]. By contrast, the S(IV)+ Fe×Mn pathway was more prevalent in summer (2.1 × 10^−5^–3.8 ×10^−2^ µg m^−3^ h^−1^) than those in other seasons (2.8 × 10^−6^–8.3 × 10^−3^ µg m^−3^ h^−1^ in autumn, 1.6 × 10^−5^–3.0 × 10^−2^ µg m^−3^ h^−1^ in winter, and 7.3 × 10^−6^–1.2 × 10^−2^ µg m^−3^ h^−1^ in spring). A previous study in Tianjin reported that secondary SO_4_^2−^ formation rates by the S(IV)+Fe×Mn pathway were higher in summer than those in other seasons [14]. Our calculated wintertime secondary SO_4_^2−^ formation rates by the S(IV)+Fe×Mn pathway were lower than those in Beijing (~4.6 µg m^−3^ h^−1^) [15] and Wangdu (10^−1^–10^0^ µg m^−3^ h^−1^) [15]. In the following section, we will systematically explain what factors impact the secondary SO_4_^2−^ formation rates by the S(IV)+H_2_O_2_ and S(IV)+Fe×Mn pathways. 

**Figure 5:**
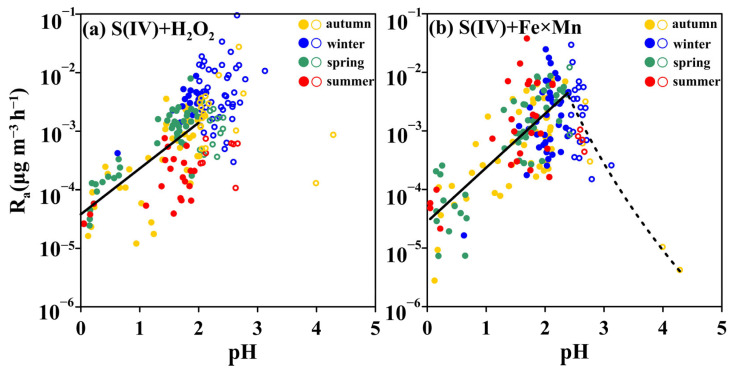
A scatter plot of aerosol pH with secondary SO_4_^2−^ formation rates by the S(IV)+H_2_O_2_ (**a**) and S(IV)+Fe×Mn (**b**) pathways. The solid circles represent pH < 2 (**a**) and pH < 2.4 (**b**), and the open circles indicate pH > 2 (**a**) and pH > 2.4 (**b**).

Different from the S(IV)+NO_2_ pathway, there were no positive or negative relationships between ionic strength and secondary SO_4_^2−^ formation rates by S(IV)+H_2_O_2_ and S(IV)+Fe×Mn pathways (Figure 4b,c), indicating that ionic strength has less of an influence on the S(IV)+H_2_O_2_ and S(IV)+Fe×Mn pathways to produce secondary SO_4_^2−^. The positive correlation between aerosol pH and secondary SO_4_^2−^ formation rates by S(IV)+H_2_O_2_ (Figure 5a) emphasizes that aerosol pH is the primary controlling factor for the S(IV)+H_2_O_2_ pathway. The chemical rate constant k_S(IV)+H2O2_ is a function of pH; when the pH value increased from 0 to 2, the chemical rate constant k_S(IV)+H2O2_ showed an increasing trend (Figure S8a). In our in situ observations, when pH < 2, the secondary SO_4_^2−^ formation rate by the S(IV)+H_2_O_2_ pathway displayed a positive relationship with the pH value (Figure 5a). This phenomenon is supported by the simultaneous increase in the chemical rate constant k_S(IV)+H2O2_ (Figure S8a) and HSO_3_^−^ concentrations (Figure S5) as pH increased from 0 to 2. In addition, our observed results showed that secondary SO_4_^2−^ formation rates by the S(IV)+H_2_O_2_ pathway under pH of 2~3 were higher than those under pH of 0~2 (Figure 5a). The influence of the chemical rate constant k_S(IV)+H2O2_ on the secondary SO_4_^2−^ formation rate can be excluded due to k_S(IV)+H2O2_ at pH 0~2 being close to those at pH 2~3 (Figure S8a). However, the percentages of HSO_3_^−^ to S(IV) at a pH of 2~3 (50–90%) were higher than those at pH of 0~2 (~50%, Figure S5). HSO_3_^−^ is a unique species of S(IV) that can react with H_2_O_2_ to form secondary SO_4_^2−^ [29], which explains the high secondary SO_4_^2−^ formation rates by the S(IV)+H_2_O_2_ pathway [10,26]. In addition, the higher pH in winter (Figure 2c) results in higher HSO_3_^−^ concentrations than in other seasons, supporting the faster SO_4_^2−^ formation rates by the S(IV)+H_2_O_2_ pathway in winter than in other seasons (Figure 5a).

The pH also has a direct influence on the secondary SO_4_^2−^ formation rate by the S(IV)+Fe×Mn pathway. pH determines not only the water-soluble concentrations of Fe(III) but also the hydrogen ion concentrations [56,57]. When pH < 2.4, there was a positive relationship between the secondary SO_4_^2−^ formation rate by the S(IV)+Fe×Mn pathway and the pH value (Figure 5b). This phenomenon can be attributed to (1) the concentrations of water-soluble Fe(III) and Mn(II) maintaining the highest levels at low pH (Figure S6), (2) the S(IV) concentrations increasing as pH increased from 0 to 2.4 (Figure S8b), and (3) the concentrations of Fe(III) and Mn(II) being influenced not only by pH but also by the AWC. Lower AWC concentrations result in higher Fe(III) and Mn(II) concentrations, favoring secondary SO_4_^2−^ formation [10,15,58]. This also explains the high rate of secondary SO_4_^2−^ formation by the S(IV)+Fe×Mn pathway in summer.

### 3.6. Comparison of Secondary SO_4_^2−^ Formation Rates under Different PM_2.5_ Levels

**Figure 6:**
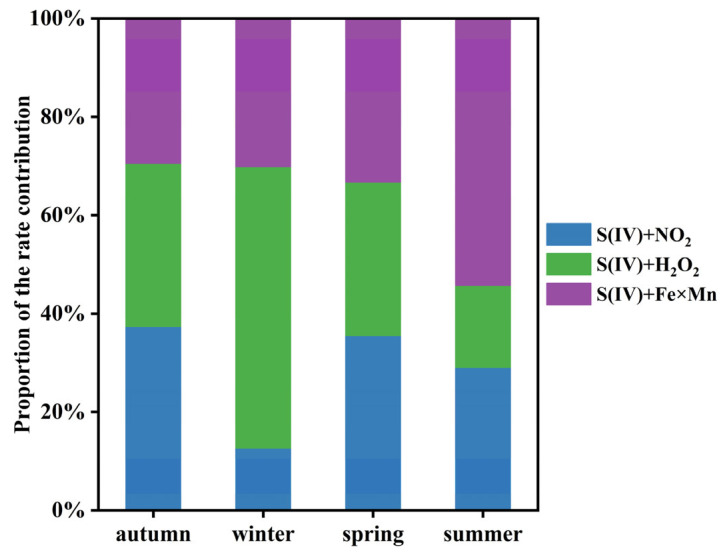
The relative contributions of S(IV)+NO_2_, S(IV)+H_2_O_2_, and S(IV)+Fe×Mn pathways to total secondary SO_4_^2−^ production.

The relative contributions of the S(IV)+NO_2_, S(IV)+H_2_O_2_, and S(IV)+Fe×Mn pathways to total secondary SO_4_^2−^ production in different seasons are shown in Figure 6. In autumn and spring, the relative contributions of each pathway were comparable. In winter, the S(IV)+H_2_O_2_ pathway was the main formation pathway, contributing to 57% of secondary SO_4_^2−^ production, but in summer, the S(IV)+Fe×Mn pathway was the largest contributor (54%). A study in Tianjin also reported that the S(IV)+H_2_O_2_ pathway played a dominant role in wintertime secondary SO_4_^2−^ production (71%), while S(IV)+Fe×Mn was the main pathway for secondary SO_4_^2−^ production in summer (55%) [14]. In addition, the contribution of the S(IV)+H_2_O_2_ and S(IV)+NO_2_ pathways to total secondary SO_4_^2−^ production had the lowest percentages (17% in summer and 13% in winter).

To understand the secondary SO_4_^2−^ aqueous-phase formation pathways in different regions under different air pollution levels in China, we compared the published datasets of secondary SO_4_^2−^ formation rates and their relative contributions in different cities and seasons (Table 1). During winter, under PM_2.5_ concentrations < 75 μg m^−3^, our observations in Haikou showed that S(IV)+H_2_O_2_ was the main secondary SO_4_^2−^ formation pathway, while a previous study in Beijing reported that S(IV)+Fe×Mn was the fastest pathway for secondary SO_4_^2−^ production [15]. The discrepancies between this study and the previous study in Beijing may be related to the selection of calculating parameters. In our observations, we measured the water-soluble concentrations of Fe and Mn and estimated the H_2_O_2_ concentrations using an empirical equation [14,30]. However, in Song et al.’s study, the authors measured H_2_O_2_ concentrations but cited the water-soluble concentrations of Fe and Mn from other studies [15]. By further comparing the calculation processes in different studies, we found that the authors of these studies did not directly measure the water-soluble concentrations of Fe and Mn; instead, they assumed concentrations [11,15,59]. In addition, secondary SO_4_^2−^ formation in Guangzhou and Zhengzhou exhibited different pathways, with these differences potentially being a result of the assumed parameters [58,60,61]. Thus, synchronous analyses of multiple chemical parameters (such as gas-phase NO_2_ and O_3_, as well as water-soluble ions) are an important basis for the accurate calculation of secondary SO_4_^2−^ formation rates during winter haze periods.

Surprisingly, a limited number of summarized datasets have shown that the S(IV)+Fe×Mn pathway is the highest contributor to secondary SO_4_^2−^ formation in summer in both southern and northern cities of China under different PM_2.5_ levels (Table 1). These results may be explained by the relatively high Fe(III) and Mn(II) concentrations due to the low pH, which promotes secondary SO_4_^2−^ formation [14,51]. In addition, a low AWC causes relatively higher water-soluble Fe(III) and Mn(II) levels, further enhancing the reactivity of the S(IV)+Fe×Mn pathway in summer [10,15]. Although secondary SO_4_^2−^ formation by the S(IV)+Fe×Mn pathway in Haikou had two to three orders of magnitude difference compared to other studies in Wangdu and Tianjin, the relative contributions of the S(IV)+Fe×Mn pathway to total secondary SO_4_^2−^ formation are comparable with Tianjin.

## 4. Conclusions

In this study, based on one-year observations of water-soluble inorganic ions, Fe, and Mn, combined with various kinetic models, we calculated the formation rate of secondary SO_4_^2−^ in Haikou and also explored the influences of ionic strength and pH on secondary SO_4_^2−^ formation aqueous-phase oxidation pathways. Our results indicated that the main secondary SO_4_^2−^ formation reactions are the S(IV)+NO_2_, S(IV)+H_2_O_2_, and S(IV)+Fe×Mn pathways for the whole year. On a seasonal scale, the S(IV)+H_2_O_2_ reaction dominated the secondary SO_4_^2−^ production rate, contributing to 57% of secondary SO_4_^2−^ formation, while the S(IV)+NO_2_ reaction only accounted for 13% of secondary SO_4_^2−^ formation in winter. In summer, the S(IV)+Fe×Mn reaction dominated the secondary SO_4_^2−^ production rate, accounting for 54% of secondary SO_4_^2−^ production. Ionic strength directly affects the chemical rate constant of k_S(IV)+NO2_, thereby controlling the secondary SO_4_^2−^ formation rates from the S(IV)+NO_2_ pathway. Aerosol pH mediates the concentrations of HSO_3_^−^ and water-soluble Fe and Mn; thus, the secondary SO_4_^2−^ formation rates by the S(IV)+H_2_O_2_ and S(IV)+Fe×Mn pathways are mainly controlled by aerosol pH. In addition, the statistical dataset showed that, in summer, the S(IV)+Fe×Mn pathway is the main reaction for secondary SO_4_^2−^ formation in China. 

## Figures and Tables

**Table 1:** Summary of the secondary sulfate formation pathway in different regions.

Regions		Sampling Time	PM_2.5_ Concentrations	Main Pathway	Mean Rate	Range	Relative Contributions	References
			(μg m^−3^)		(μg m^−3^ h^−1^)	(μg m^−3^ h^−1^)	(%)	
Haikou	Winter	December 2021–February 2022	6.2–52	S(IV)+H_2_O_2_	7.2 × 10^−3^	3.0 × 10^−4^–9.5 × 10^−2^	57	this study
Beijing		December 2017	18 ± 10–52 ± 10	S(IV)+Fe×Mn	^−^	0.9–1.0 ^a^	^−^	[15]
Beijing		January 2013	>75(haze)	S(IV)+NO_2_	^−^	1–7^a^	^−^	[11]
Beijing		January 2016	>75(haze)	S(IV)+H_2_O_2_	^−^	~2.7 × 10^−1 a^	^−^	[59]
Beijing		December 2017	>75(haze)	S(IV)+Fe×Mn	^−^	1.0–1.8 ^a,b^	^−^	[15]
Guangzhou		2005	haze-fog	S(IV)+NO_2_	^−^	1.0–6.4	^−^	[60]
Zhengzhou		January2020–February 2020	>75(haze)	S(IV)+Fe×Mn	^−^	2.0 × 10^−2^–1.2 × 10^−1^	^−^	[61]
Zhengzhou		January 2018	>75(haze)	S(IV)+Fe×Mn	^−^	10^−1^–10^0 a^	^−^	[58]
Xinxiang		January 2018	>75(haze)	S(IV)+H_2_O_2_	10^−1 a^	^−^		[58]
Haikou	Summer	June 2022–August 2022	3.9–15	S(IV)+Fe×Mn	3.7 × 10^−3^	2.1 × 10^−5^–3.8 × 10^−2^	54	this study
Wangdu		June 2014	20 ± 10–55 ± 12	S(IV)+Fe×Mn	^−^	1.2–2.3 ^a,b^	^−^	[15]
Wangdu		June 2014	>75(haze)	S(IV)+Fe×Mn	^−^	1.9–3.6 ^a,b^	^−^	[15]
Tianjin		June 2018–August 2018, June 2019–August 2019	>75(haze)	S(IV)+Fe×Mn	^−^	^−^	55	[14]

^a^ The values are obtained from the graph. ^b^ Recalculation based on original data.

## Data Availability

Data are contained within the article and Supplementary Materials.

## Supplementary Materials

The following supporting information can be downloaded at: https://www.mdpi.com/article/10.3390/toxics12020105/s1, Figure S1: The schematic of sulfate formation pathways, Figure S2: Sampling location, Figure S3: Hourly concentrations of T, RH, PM_2.5_, PM_10_, SO_2_, NO_2_, O_3_, and CO from September 2021 to August 2022 (The yellow, blue, green, and red bars represent autumn, winter, spring, and summer, respectively), Figure S4: Comparisons of H_2_O_2_ concentrations at different sites in different seasons, Figure S5: Compositions of S(IV) species with regard to pH, Figure S6: Water-soluble Fe(III) and Mn(II) concentrations as a function of pH, Figure S7: Influence of ionic strength (I) on chemical rate constant k_S(IV)+NO2_ (k^I=i^ is the rate constant at I = i M, k^I=0^ is the rate constant at I=0 M, Figure S8: (a) Second-order rate constant for oxidation of S(IV) by H_2_O_2_ as a function of pH, (b) Effective Henry’s law constant (H*_S(IV)_) for SO_2_ as a function of pH, Table S1: Rate constants for calculating Henry’s law, Table S2: Rate constants and ionic strength effects of the reactions, Table S3: The mean concentrations and ranges of water-soluble inorganic ions (in unit μg m^−3^) in different seasons [62,63,64,65,66,67,68,69,70,71,72,73,74,75].
