# Supplementary material for: Assessment of Secondary Sulfate Aqueous-Phase Formation Pathways in the Tropical Island City of Haikou: A Chemical Kinetic Perspective
Source: Toxics. 2024 Jan 26;12(2):105. doi: 10.3390/toxics12020105 (PMC10892436; doi:10.3390/toxics12020105)
Supplement: Supplementary file 1 [file toxics-12-00105-s001.zip › toxics-2799045-supplementary.pdf]

# Supplementary Materials

## Assessment of Secondary Sulfate Aqueous-Phase Formation Pathways in the Tropical Island City of Haikou: A Chemical Kinetic Perspective

Text S1

$H_{SO2}$, $K_{s1}$ and $K_{s2}$ can be estimated by Equations (S1) and (S2) [62],

$$H = H(298K)exp\left[-\frac{\Delta H_{298K}}{R}\left(\frac{1}{T}-\frac{1}{T_{298K}}\right)\right] \quad \text{(S1)}$$

where $\Delta H_{298K}$ is the enthalpy change at constant temperature and pressure; the parameters of $\Delta H_{298k}/R$ are shown in Table S1; $T$ indicates the absolute temperature (K); and $H_{SO2}$(298K) = 1.23 M atm$^{-1}$ (Table S1).

$$\mathrm{Ks} = K(298K)exp\left[-\frac{\Delta H_{298K}}{R}\left(\frac{1}{T}-\frac{1}{T_{298K}}\right)\right] \quad \text{(S2)}$$

where $\Delta H_{298K}$ is the enthalpy change at constant temperature and pressure; the parameters of $\Delta H_{298k}/R$ are −1960 and −1500, respectively; $T$ indicates the absolute temperature (K); and $K$(298K) are 1.3 ×10$^{-2}$ M for $K_{s1}$ and 6.6 ×10$^{-8}$ M for $K_{s2}$.

Text S2

The influences of ionic strength on the reaction rate of the $S(IV)+NO_2$ pathway can be estimated by Equation (S3) [11,63],

$$log\frac{k_{S(IV)+NO_2}{}^{I=i}}{k_{S(IV)+NO_2}{}^{I=0}} = b\ I_i \qquad (S3)$$

where $k_{S(IV)+NO_2}{}^{I=i}$ is the rate constant at I=i (M); $I_i$ is the ionic strength, which was calculated by the ISORROPIA II model; $k_{S(IV)+NO_2}{}^{I=0}$ ($M^{-1}$ $s^{-1}$) represents the rate constant at I=0 M; and *b* is the kinetic salting coefficient, taken as 0.5 [11].

Text S3

The influences of ionic strength on the reaction rate of the $S(IV)+H_2O_2$ pathway can be estimated by Equation (S4) [64],

$$log\frac{{k_{S(IV)+H2O_2}}^{I=i}}{{k_{(S(IV)+H_2O_2)}}^{I=0}} = -\frac{2\times0.509\times\sqrt{I_i}}{1+0.17\times\sqrt{I_i}} + 2\times0.18I_i \quad \text{(S4)}$$

where ${k_{S(IV)+H_2O_2}}^{I=i}$ ($M^{-1}$ $s^{-1}$) is the rate constant at I=i (M); $I_i$ is the ionic strength, which was calculated by the ISORROPIA II model; and ${k_{S(IV)+H_2O_2}}^{I=0}$ ($M^{-1}$ $s^{-1}$) represents the rate constant at I=0 M.

Text S4

The influences of ionic strength on the reaction rate of the $S(IV)+O_3$ pathway can be estimated by Equation (S5) [65],

$$\frac{{k_{S(IV)+O_3}}^{I=i}}{{k_{S(IV)+O_3}}^{I=0}} = 1 + 1.94 I_i \quad \text{(S5)}$$

where ${k_{S(IV)+O_3}}^{I=i}$ ($M^{-1}$ $s^{-1}$) is the rate constant of the $S(IV)+O_3$ pathway at I=i (M); $I_i$ is the ionic strength, which was calculated by the ISORROPIA II model; and ${k_{S(IV)+O_3}}^{I=0}$ ($M^{-1}$ $s^{-1}$) represents the rate constant at I=0 M.

Text S5

The rate expression of Fe(III)-catalyzed oxidation of S(IV) into secondary $SO_4^{2-}$ varied with aerosol pH [26,42,66,67],

pH 0 to 3:

$$R_{aq(Fe+O_2)} = k_{(Fe+O_2)1}[H^+]^{-1}[Fe(III)][S(IV)] \quad \text{(S6)}$$

pH 3 to 4.5:

$$R_{aq(Fe+O_2)} = k_{(Fe+O_2)2}[Fe(III)]^2[S(IV)] \quad \text{(S7)}$$

pH 4.5 to 6.5:

$$R_{aq(Fe+O_2)} = k_{(Fe+O_2)3}[S(IV)] \quad \text{(S8)}$$

pH > 6.5:

$$R_{aq(Fe+O_2)} = k_{(Fe+O_2)4}[S(IV)] \quad \text{(S9)}$$

where $k_{(Fe+O_2)1}$ = 6 $s^{-1}$, $k_{(Fe+O_2)2}$ = $1.0\times10^9$ $M^{-2}$ $s^{-1}$, $k_{(Fe+O_2)3}$ =$1.0\times10^{-3}$ $s^{-1}$ and $k_{(Fe+O_2)4}$ =$1.0\times10^{-4}$ $s^{-1}$ [26,42] (Table S2); [$H^+$] is $H^+$ concentration and calculated by the ISORROPIA II model; and [Fe(III)] is the concentrations of Fe(III) in aerosol water, which was calculated by Wang T et al. [10]:

$$[Fe(III)] = Min\left(C_{Fe} \times F/M_{Fe}/AWC, \frac{K_{sp,Fe(OH)_3}}{[OH^-]^3}\right) \quad \text{(S10)}$$

where $C_{Fe}$ (ng $m^{-3}$) is the concentration of water-soluble Fe; $F$ is the fraction of Fe(III) in water-soluble Fe, which is 10% during the day and 90% at night [68]; $M_{Fe}$ (g $mol^{-1}$) is the molecular weight of Fe; AWC is the aerosol water content in L $m^{-3}$ and was calculated by the ISORROPIA II model; and $K_{sp,Fe(OH)_3}$ represents the solubility product constant of $Fe(OH)_3$, which was taken as $6\times10^{-38}$ M [69].

The influences of ionic strength on the reaction rate of the S(IV)+Fe pathway

can be estimated by Equation (S11) [10],

$$log\frac{k_{(Fe+O_2)}{}^{I=i}}{k_{(Fe+O_2)}{}^{I=0}} = -4\frac{\sqrt{I_i}}{1+\sqrt{I_i}} \quad (S11)$$

where $k_{Fe+O_2}{}^{I=i}$ is the rate constant of the S(IV)+Fe pathway at I=i (M); $I_i$ is the ionic strength, which was calculated by the ISORROPIA II model; and $k_{Fe+O_2}{}^{I=0}$ represents the rate constant at I=*0* M.

The rate expression for the Mn(II)-catalyzed oxidation reaction is zero-order kinetics, for the S(IV) concentrations above 100 μM [70],

$$R_{aq(Mn+O_2)} = k_{(Mn+O_2)1}[Mn(II)]^2 \quad (S12)$$

For S(IV) concentration below 1 μM, the reaction obeys first order in S(IV) [70],

$$R_{aq(Mn+O_2)} = k_{(Mn+O_2)2}[Mn(II)][S(IV)] \quad (S13)$$

where $k_{(Mn+O_2)1}$ = 680 $M^{-1}$ $s^{-1}$; $k_{(Mn+O_2)2}$ = 1000 $M^{-1}$ $s^{-1}$ [26]; and [Mn(II)] is the concentration of Mn(II) in aerosol water, which was calculated by Wang T et al. [10]:

$$[Mn(II)] = Min\left(C_{Mn}/M_{Mn}/AWC, \frac{K_{sp,Mn(OH)_2}}{[OH^-]^2}\right) \quad (S14)$$

where $C_{Mn}$ (ng $m^{-3}$) is the concentration of water-soluble Mn; $M_{Mn}$ (g $mol^{-1}$) is the molecular weight of Mn; AWC is the aerosol water content in L $m^{-3}$, which was calculated by the ISORROPIA II model; and $K_{sp,Mn(OH)_2}$ represents the solubility product constant of $Mn(OH)_2$, which is $1.6\times10^{-13}$ M [69].

The influences of ionic strength on the reaction of the S(IV)+Mn pathway can be estimated by Equation (S15) [26],

$$log\frac{k_{(Mn+O_2)}{}^{I=i}}{k_{(Mn+O_2)}{}^{I=0}} = -4.07\frac{\sqrt{I_i}}{1+\sqrt{I_i}} \quad (S15)$$

where $k_{Mn+O_2}^{I=i}$ ($M^{-1}$ $s^{-1}$) is the rate constant of the S(IV)+Mn pathway at I=i (M); $I_i$ is the ionic strength, which was calculated by the ISORROPIA II model; and $k_{Mn+O_2}^{I=0}$ ($M^{-1}$ $s^{-1}$) represents the rate constant at I=0 M.

Text S6

The influences of ionic strength on the reaction rate of the S(IV)+Fe×Mn pathway can be estimated by Equation (S16) [10],

$$log\frac{{k_{(Fe\times Mn+O_2)}}^{I=i}}{{k_{(Fe\times Mn+O_2)}}^{I=0}} = -4\frac{\sqrt{I_i}}{1+\sqrt{I_i}} \quad \text{(S16)}$$

where $k_{Fe\times Mn+O_2}{}^{I=i}$ ($M^{-2}$ $s^{-1}$) is the rate constant of the S(IV)+Fe×Mn pathway when I=$i$ (M); $I_i$ is the ionic strength, which was calculated by the ISORROPIA II model; and $k_{Fe\times Mn+O_2}{}^{I=0}$ ($M^{-2}$ $s^{-1}$) represents the rate constant at I=0 M.

Text S7

$J_{aq,lim} = \min\{J_{aq}(SO_2), J_{aq}(O_{xi})\}$ (S17)

where $J_{aq}(O_{xi})$ refers to the limiting mass transfer rate of the oxidants ($NO_2$, $H_2O_2$, $O_3$).

$J_{aq}(X) = K_{MT}(X) \cdot p(X) \cdot H(X)$ (S18)

where X represents the $SO_2$ or the other oxidants ($NO_2$, $H_2O_2$, $O_3$); $p$(X) is the gas-phase partial pressure (atm); $H$(X) is the Henry's law constant ($M^{-1}$ $atm^{-1}$); and $K_{MT}$ is the mass transfer rate coefficient ($s^{-1}$), and can be calculated as:

$K_{MT}(X) = \left[\frac{R_P^2}{3D_g} + \frac{4R_p}{3\alpha v}\right]^{-1}$ (S19)

where $\frac{R_P^2}{3D_g}$ and $\frac{4R_p}{3\alpha v}$ refer to the continuum regime resistance and the kinetic (or free-molecular) regime resistance, respectively. $R$p is the radius of aerosol (m), which we assumed as $1.5\times10^{-7}$ m. $D$g ($m^2$ $s^{-1}$) is the gas-phase molecular diffusion coefficient, and we adopted the values from the literature as $2\times10^{-5}$ [14]. $A$ is the mass accommodation coefficient of $X$ on the droplet surface: for $SO_2$, it is 0.11; for $O_3$, $5\times10^{-4}$; for $H_2O_2$, 0.23; and for $NO_2$, $2\times10^{-4}$ [26,71]. $v$ (m $s^{-1}$) is the mean molecular speed of X.

Table S1. Rate constants for calculating Henry's law.

| Gas species | Equilibrium | Constants (298K) (M atm$^{-1}$) | $\Delta H_{298k}/R$(K) | Reference |
|---|---|---|---|---|
| $SO_2$ | $SO_2(g) \leftrightarrow SO_2(aq)$ | $H_{SO2}$=1.23 | −3145.3 | [26] |
| $NO_2$ | $NO_2(g) \leftrightarrow NO_2(aq)$ | $H_{NO2}$=1.0×10$^{-2}$ | −2516.2 | |
| $H_2O_2$ | $H_2O_2(g) \leftrightarrow H_2O_2(aq)$ | $H_{H2O2}$=1.0×10$^{5}$ | −7297.1 | |
| $O_3$ | $O_3(g) \leftrightarrow O_3(aq)$ | $H_{O3}$=1.1×10$^{-2}$ | −2536.4 | |

Table S2. Rate constants and ionic strength effects of the reactions.

| Oxidants | Rate constants | E/R(K) | I impact | References |
|---|---|---|---|---|
| $NO_2$ | $k_{S(IV)+NO_2,low} = (0.14\sim2) \times 10^6 (M^{-1}\ s^{-1})$<br>$k_{S(IV)+NO_2,high} = (1.24\sim1.67) \times 10^7 (M^{-1}\ s^{-1})$ | – | $log\frac{k}{k^{I=0}} = 0.5I$ | [11,27,28] |
| $H_2O_2$ | $k_{(S(IV)+H_2O_2)1}(298K) = 7.45 \times 10^7 (M^{-1}\ s^{-1})$ | 4430 | $log\frac{k}{k^{I=0}} = -\frac{2 \times 0.509\sqrt{I}}{1 + 0.17 \times \sqrt{I}} + 2 \times 0.18I$ | [26,29,64] |
| $O_3$ | $k_{(S(IV)+O_3)1} = 2.4 \times 10^4 (M^{-1}\ s^{-1})$ | – | $\frac{k}{k^{I=0}} = 1 + 1.94I$ | [26,65] |
| | $k_{(S(IV)+O_3)2}(298K) = 3.7 \times 10^5 (M^{-1}\ s^{-1})$ | 5530 | | |
| | $k_{(S(IV)+O_3)3}(298K) = 1.5 \times 10^9 (M^{-1}\ s^{-1})$ | 5280 | | |
| Fe | pH<3 $k_{(Fe+O_2)1} = 6(s^{-1})$<br>3<pH<4.5 $k_{(Fe+O_2)2} = 1.0 \times 10^9 (M^{-2}\ s^{-1})$<br>4.5<pH<6.5 $k_{(Fe+O_2)3} = 1.0 \times 10^{-3} (s^{-1})$<br>pH>6.5 $k_{(Fe+O_2)4} = 1.0 \times 10^{-4} (s^{-1})$ | – | $log\frac{k}{k^{I=0}} = -4\frac{\sqrt{I}}{1+\sqrt{I}}$ | [10,66,67] |
| Mn | S(IV)>100μM $k_{(Mn+O_2)1} = 680 (M^{-1}\ s^{-1})$<br>S(IV)<1μM $k_{(Mn+O_2)2} = 1000 (M^{-1}\ s^{-1})$ | – | $log\frac{k}{k^{I=0}} = -4.07\frac{\sqrt{I}}{1+\sqrt{I}}$ | [26,70] |
| Fe×Mn | pH≤4.2 $k_{(Fe\times Mn+O_2)1}(297K) = 3.72 \times 10^7 (M^{-2}\ s^{-1})$<br>pH≥4.2 $k_{(Fe\times Mn+O_2)2}(297K) = 2.51 \times 10^{13} (M^{-2}\ s^{-1})$ | 8430 | $log\frac{k}{k^{I=0}} = -4\frac{\sqrt{I}}{1+\sqrt{I}}$ | [26,31] |

Table S3. The mean concentrations and ranges of water-soluble inorganic ions ($\mu g\ m^{-3}$) in different seasons.

| Species | Autumn | Winter | Spring | Summer |
|---|---|---|---|---|
| nss-$SO_4^{2-}$ | 2.9±1.8 | 4.1±1.8 | 3.9±2.1 | 1.5±0.7 |
| | 0.1-8.6 | 1.1-9.2 | 1.0-13 | 0.7-4.1 |
| ss-$SO_4^{2-}$ | 0.1±0.04 | 0.1±0.1 | 0.1±0.04 | 0.04±0.02 |
| | 0.02-0.2 | 0.02-0.3 | 0.02-0.2 | 0.02-0.1 |
| $NO_3^-$ | 0.4±0.7 | 3.2±3.0 | 1.0±1.1 | 0.5±0.3 |
| | 0.1-4.3 | 0.4-13 | 0.2-6.0 | 0.1-1.3 |
| $NH_4^+$ | 0.7±0.6 | 1.8±1.3 | 1.1±0.7 | 0.4±0.3 |
| | 0.1-2.4 | 0.3-5.9 | 0.2-3.4 | 0.1-1.3 |
| $Ca^{2+}$ | 0.3±0.4 | 0.2±0.1 | 0.3±0.2 | 0.3±0.1 |
| | 0.1-2.4 | 0.03-0.5 | 0.02-0.8 | 0.1-0.6 |
| $Na^+$ | 0.3±0.2 | 0.4±0.2 | 0.3±0.1 | 0.2±0.1 |
| | 0.1-0.9 | 0.1-1.0 | 0.1-0.7 | 0.1-0.3 |
| $Cl^-$ | 0.2±0.2 | 0.7±0.3 | 0.3±0.2 | 0.2±0.1 |
| | 0.02-1.1 | 0.2-1.7 | 0.03-1.0 | 0.03-0.5 |
| $K^+$ | 0.1±0.1 | 0.2±0.2 | 0.1±0.1 | 0.1±0.04 |
| | 0.03-0.6 | 0.1-0.8 | 0.03-0.3 | 0.02-0.2 |
| $Mg^{2+}$ | 0.04±0.02 | 0.1±0.03 | 0.1±0.02 | 0.03±0.01 |
| | 0.01-0.1 | 0.02-0.2 | 0.01-0.1 | 0.01-0.1 |

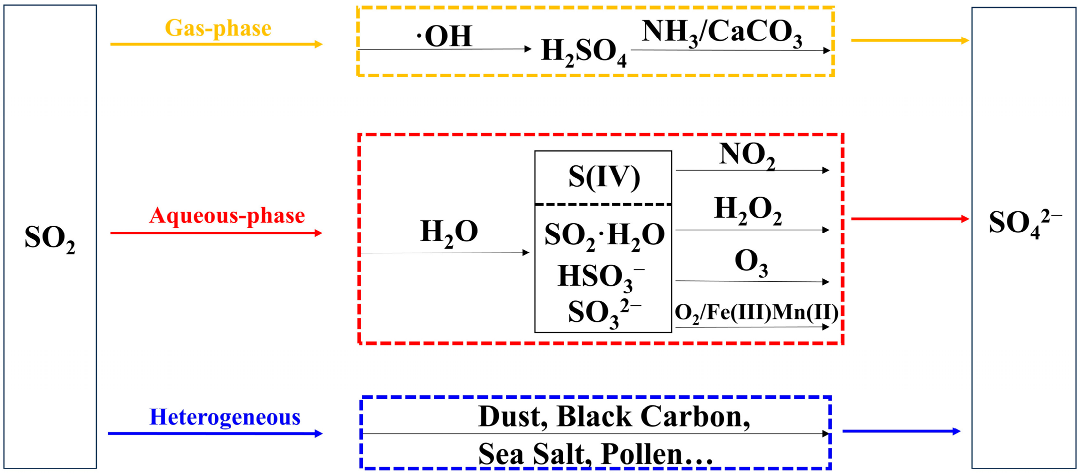


Figure S1. The schematic of sulfate formation pathways.

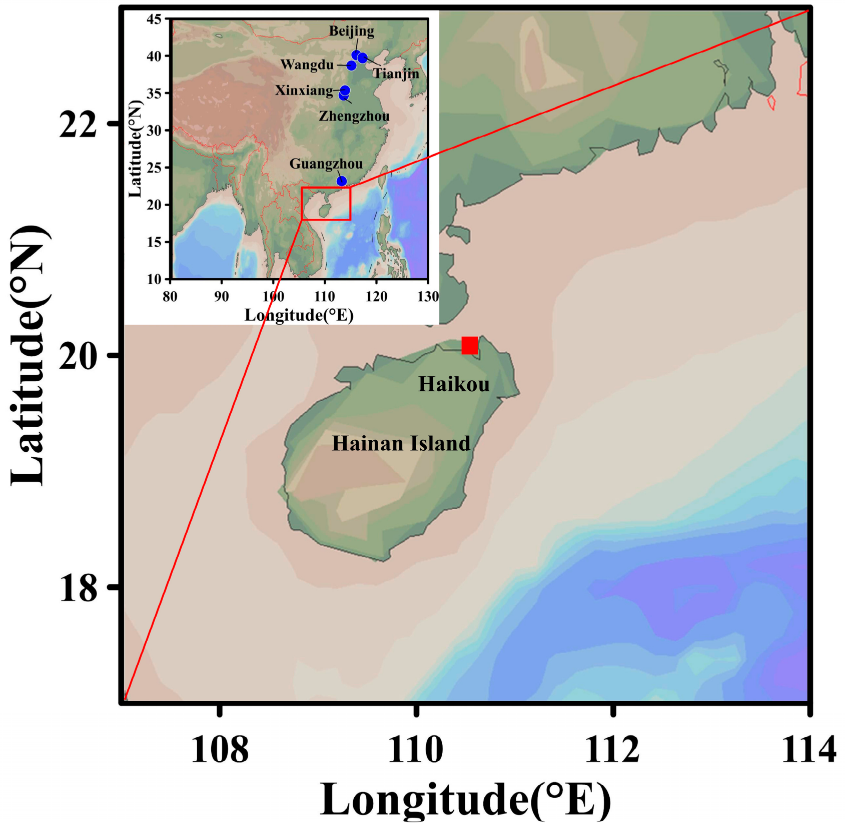


Figure S2. Sampling location.

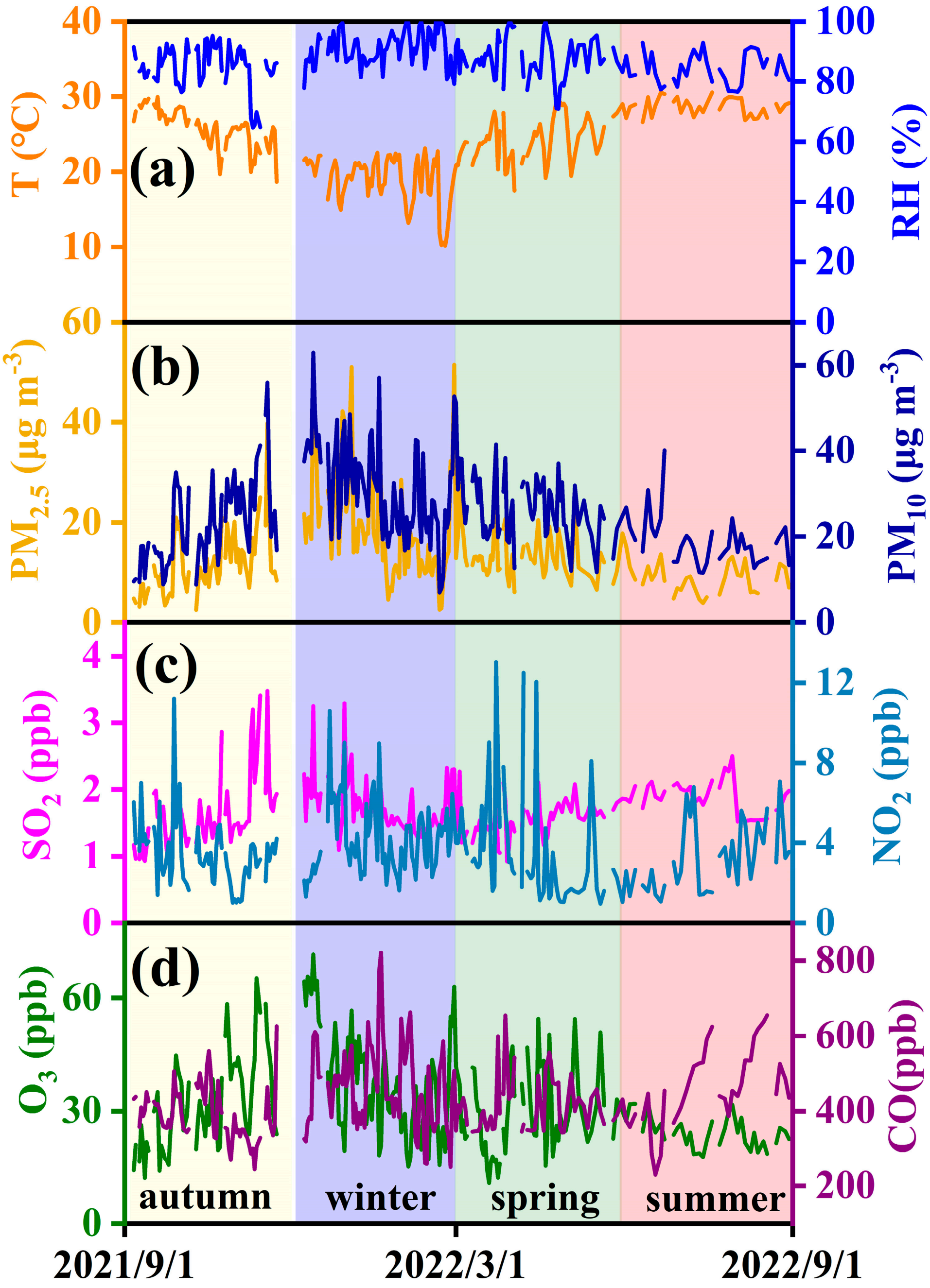


Figure S3. Hourly concentrations of T, RH, $PM_{2.5}$, $PM_{10}$, $SO_2$, $NO_2$, $O_3$, and CO from September 2021 to August 2022. (The yellow, blue, green, and red bars represent autumn, winter, spring, and summer, respectively).

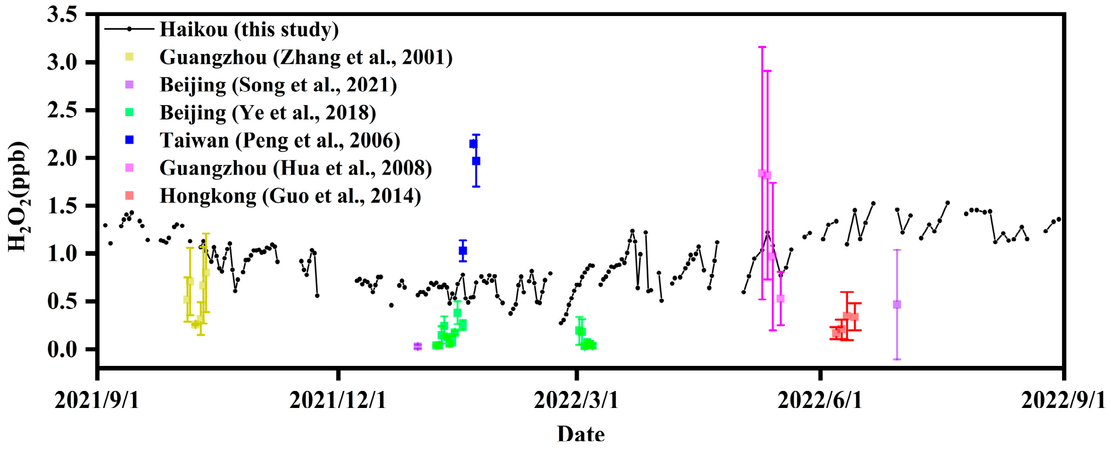


Figure S4. Comparisons of $H_2O_2$ concentrations at different sites in different seasons.

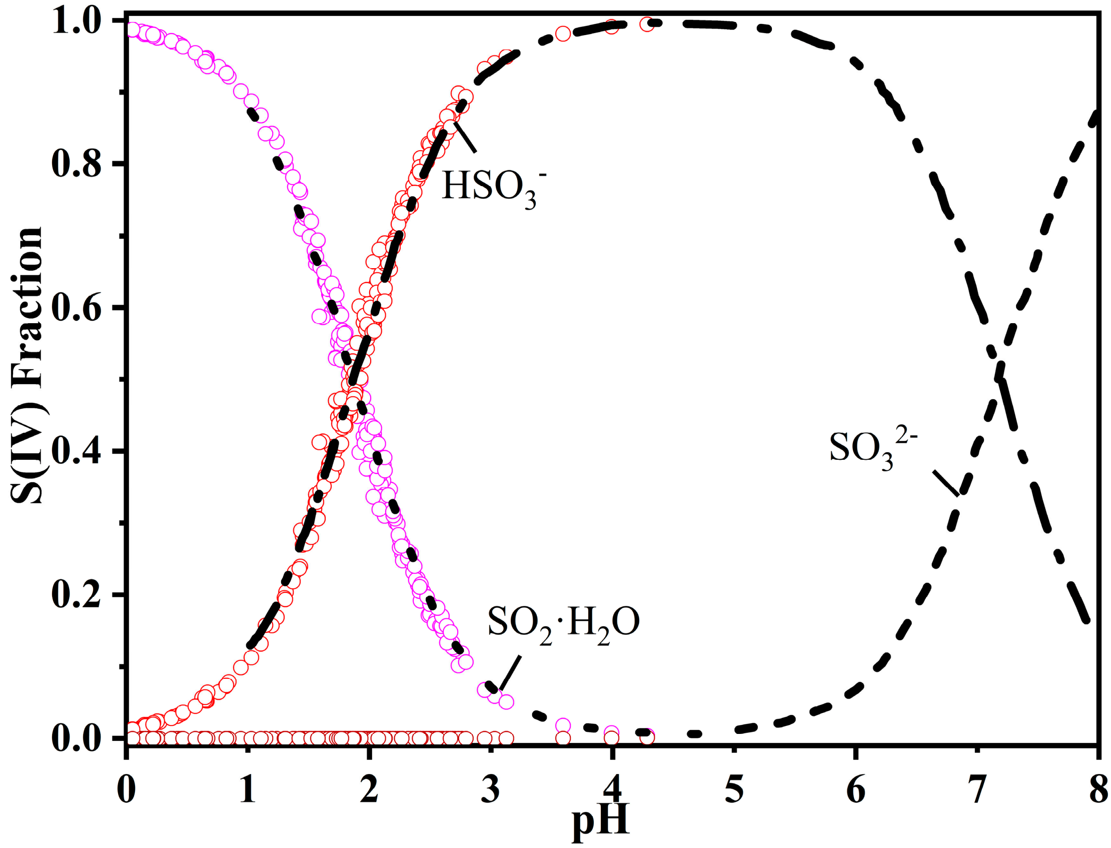


Figure S5. Compositions of S(IV) species with regard to pH. (The different circles indicate the calculated forms of S(IV) using Henry's law. The black dotted lines represent previous results in Seinfeld and Pandis [26].)

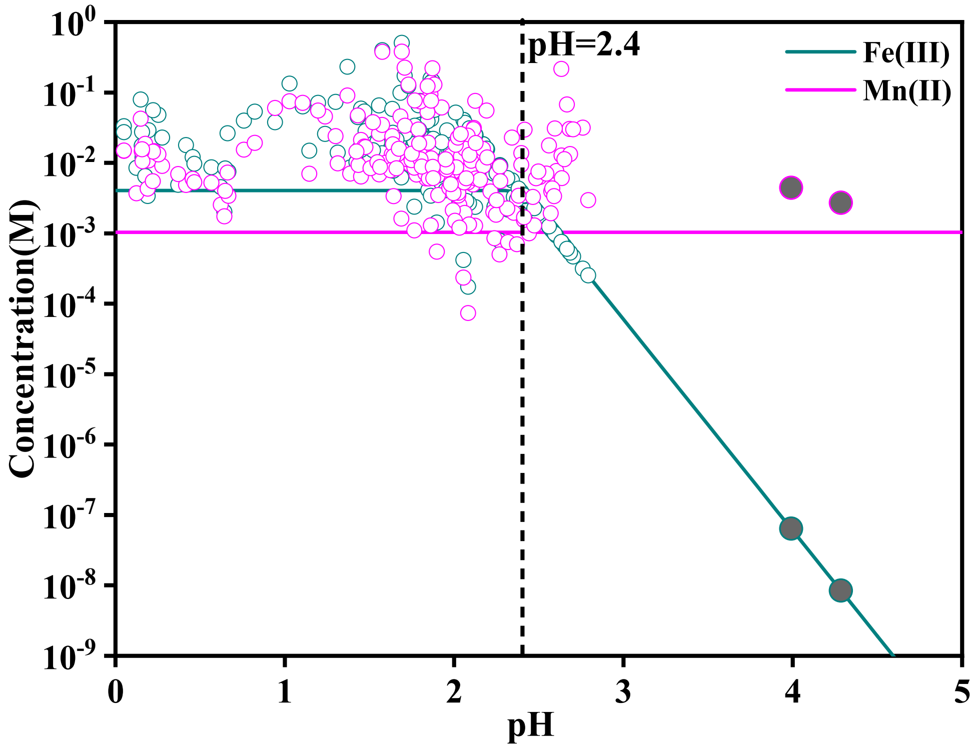


Figure S6. Water-soluble Fe(III) and Mn(II) concentrations as a function of pH. (The solid cyan and purple lines indicate the concentrations of Fe (III) and Mn (II) calculated using the mean values, respectively. The different circles indicate the actual calculated concentrations of Fe(III) and Mn(II). The vertical dashed line indicates pH = 2.4).

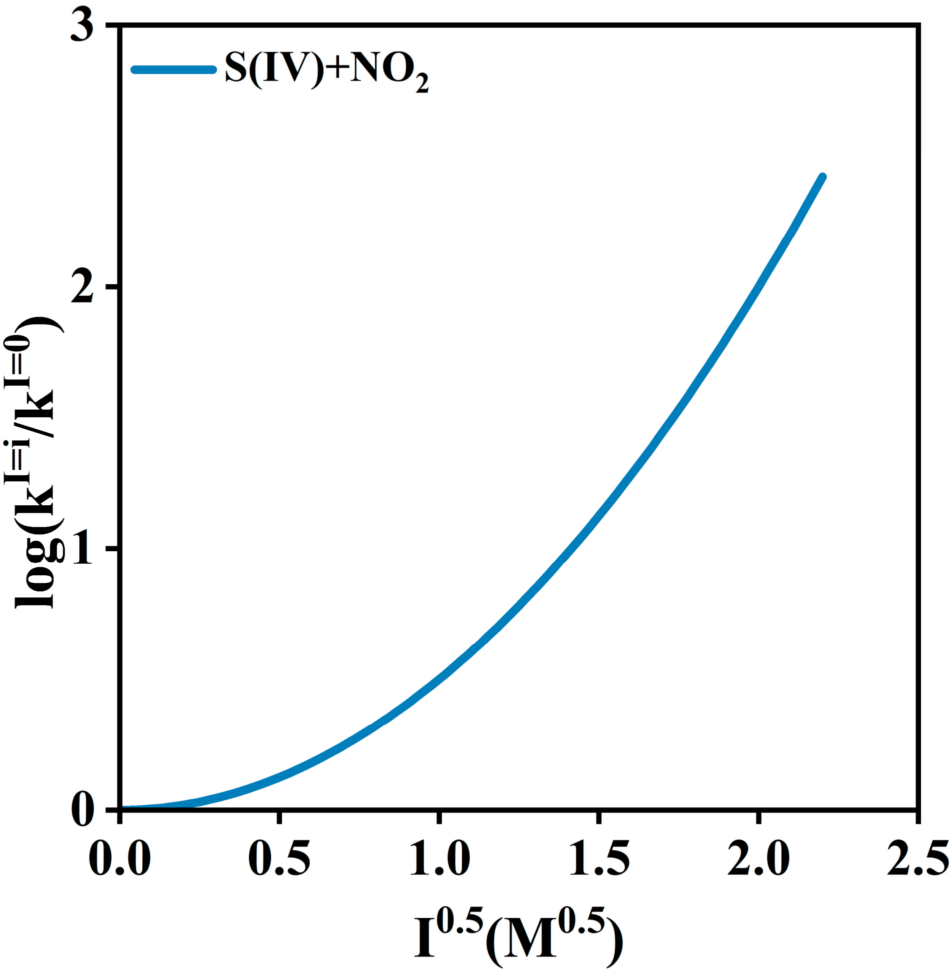


Figure S7. Influence of ionic strength (I) on chemical rate constant $k_{S(IV)+NO2}$. ($k^{I=i}$ is the rate constant at I=i (M), and $k^{I=0}$ is the rate constant at I=0 M. Detailed expressions for the $\log(k^{I=i}/k^{I=0})$ versus I relationship are shown in Table S2.)

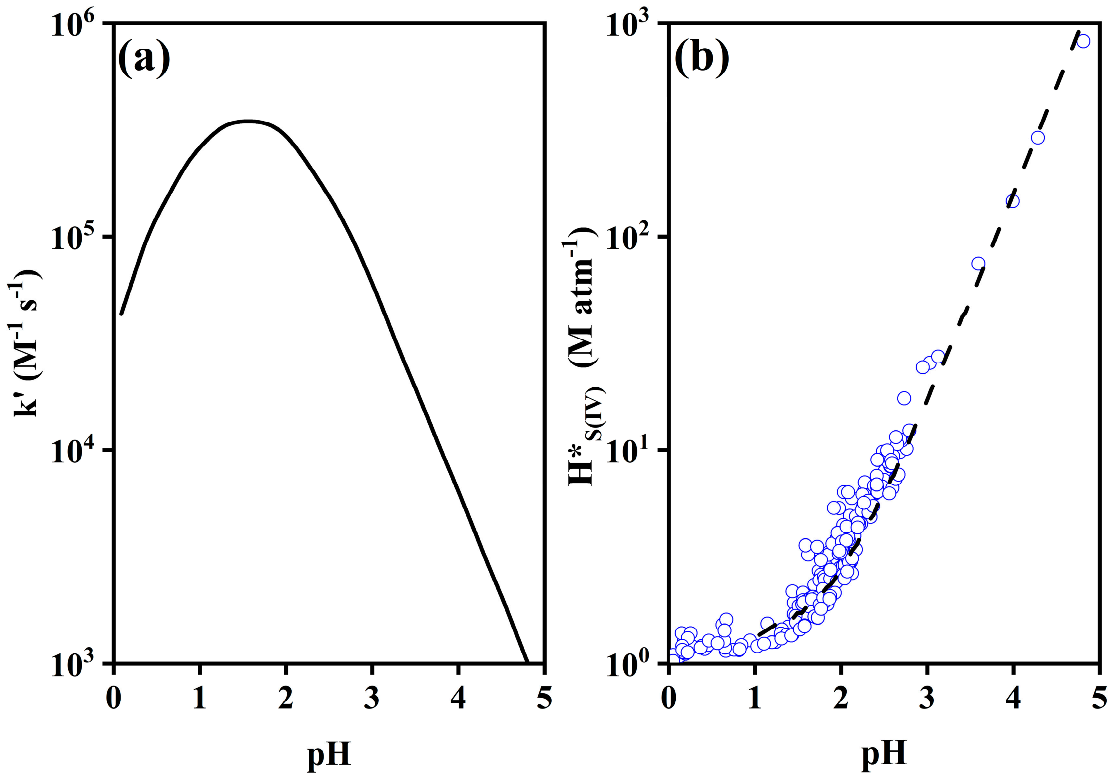


Figure S8. (a) Second-order rate constant for oxidation of S(IV) by $H_2O_2$ as a function of pH. The line is quoted from Seinfeld and Pandis [26]. (b) Effective Henry's law constant ($H^*_{S(IV)}$) for $SO_2$ as a function of pH. The black dotted line is quoted from Seinfeld and Pandis [26].

## References


62. Denbigh, K. *The principles of chemical equilibrium: with applications in chemistry and chemical engineering*, 4rd ed.; Cambridge University Press, **1981**. https://doi.org/10.1021/ja01593a082.
63. Herrmann, H. Kinetics of aqueous phase reactions relevant for atmospheric chemistry. Chemical Reviews. **2003**, 103(12), 4691-4716. https://doi.org/10.1021/cr020658q.
64. Maaß, F.; Elias, H.; Wannowius, K. Kinetics of the oxidation of hydrogen sulfite by hydrogen peroxide in aqueous solution: ionic strength effects and temperature dependence. Atmospheric Environment. **1999**, 33(27), 4413-4419. https://doi.org/10.1016/S1352-2310(99)00212-5.
65. Lagrange, J.; Pallares, C.; Lagrange, P. Electrolyte effects on aqueous atmospheric oxidation of sulphur dioxide by ozone. Journal of Geophysical Research: Atmospheres. **1994**, 99(D7), 14595-14600. https://doi.org/10.1029/94JD00573.
66. Martin, L.; Hill, M. The iron catalyzed oxidation of sulfur: Reconciliation of the literature rates. Atmospheric Environment (1967). **1967**, 21(6), 1487-1490. https://doi.org/10.1016/0004-6981(67)90100-X.
67. Martin, L.; Hill, M.; Tai, A.; Good, T. The iron catalyzed oxidation of sulfur(IV) in aqueous solution: Differing effects of organics at high and low pH. Journal of Geophysical Research: Atmospheres. **1991**, 96(D2), 3085-3097. https://doi.org/10.1029/90JD02611.
68. Siefert, R.; Johansen.; Hoffmann, M.; Pehkonen, S. Measurements of trace metal (Fe, Cu, Mn, Cr) oxidation states in fog and stratus clouds. Journal of the Air & Waste Management Association. **1998**, 48(2), 128-143. https://doi.org/10.1080/10473289.1998.10463659.
69. Graedel, T.; Weschler, C. Chemistry within aqueous atmospheric aerosols and raindrops. Reviews of Geophysics. **1981**, 19(4), 505-539. https://doi.org/10.1029/RG019i004p00505.
70. Martin, L.; Hill, M. The effect of ionic strength on the manganese catalyzed oxidation of sulfur(IV). Atmospheric Environment (1967). **1987**, 21(10), 2267-2270. https://doi.org/10.1016/0004-6981(87)90361-1.
71. Jacob, D. Heterogeneous chemistry and tropospheric ozone. Atmospheric Environment. **2000**, 34(12-14), 2131-2159. https://doi.org/10.1016/S1352-2310(99)00462-8.
72. Zhang, Y.; Ma, Y.; Zeng, L.; Shao, K.; Qi, B. Study of atmospheric peroxides in Guangzhou city. China Environmental Science. **2001**, 21(3), 221-225 (in Chinese).
73. Peng, Y.; Chen, K.; Lai, C.; Lu, P.; Kao, J. H. Concentrations of H2O2 and HNO3 and O3–VOC–NOx sensitivity in ambient air in southern Taiwan. Atmospheric Environment. **2006**, 40(35), 6741-6751. https://doi.org/10.1016/j.atmosenv.2006.05.079.
74. Hua, W.; Chen, Z.; Jie, C.; Kondo, Y.; Hofzumahaus, A.; Takegawa, N.; Chang, C.; Lu, K.; Miyazaki, Y.; Kita, K.; Wang, H.; Zhang, Y.; Hu, M. Atmospheric hydrogen peroxide and organic hydroperoxides during PRIDE-PRD'06, China: their concentration, formation mechanism and contribution to secondary aerosols. Atmospheric Chemistry and Physics. **2008**, 8(22), 6755-6773. https://doi.org/10.5194/acp-8-6755-2008.
75. Guo, J.; Tilgner, A.; Yeung, C.; Wang, Z.; Louie, P.; Luk, C.; Xu, Z.; Yuan, C.; Gao, Y.; Poon, S.; Herrmann, H.; Lee, S.; Lam, K.; Wang, T. Atmospheric peroxides in a polluted subtropical environment: seasonal variation, sources and sinks, and importance of heterogeneous processes. Environmental Science & Technology. **2014**, 48(3), 1443-1450. https://doi.org/10.1021/es403229x.
